# Correspondence

**Published:** 1860-11

**Authors:** 


### Correspondence.

Messrs. Editors:—After a somewhat protracted silence, occasioned by summer jauntings, I will, with your permission, renew my correspondence. And first, I wish to make some remarks on the late meeting of the "American Dental Association" held at Washington, as the smoke has cleared away and we can now look calmly at the results, and this I wish to do without being justly chargeable with censoriousness, or give the slightest occasion for offense; and in the hope that my interest in the success of this important movement will be sufficient apology for any strictures that may be made.

The profession were led to expect,—as it was unquestionably the purpose of its founders—that this organization, from its select, representative character, must produce results of greater moment; that its proceedings would be characterized by a more elevated tone; that it would bring out a higher order of merit, and that in a word, it should be a decided improvement in every essential feature, upon any other organization now, or that ever had been connected with the dental profession. Am I not right? Was not this the implied, if not expressed intention and purpose of its projectors?

Now, to what extent has this been accomplished? To "this extent, no more." *Four essays, and the synopsis of a fifth*, constitute the *sole product* of the meeting.

Now, it comes properly within the scope of this discussion to canvass the character of these essays, as they are essential to the argument; but as by so doing I might be charged with personalities (which above all things I wish to avoid), I forbear; and as they have all been placed before the profession, their respective and collective merits may be ascertained and determined. But I submit that, collectively, they are not up to what the profession had an undoubted right to expect, considering the character and abilities of some of the projectors and participants in this movement; and above all, that the result has not been commensurate with the avowed purposes and pretensions of its friends, and therefore a general disappointment is the result.

My hope is,—and it is a reasonable hope,—that the next annual meeting will exhibit a decided improvement upon the last, and that the interest will be enhanced by the introduction of intelligent discussions.

The committees—an extensive number of which were created—will, I take it for granted, accomplish something important, else why their appointment? But where the matter is to come from, upon which so many are to pass judgment, or whether they are to originate and prepare it themselves, or whether by report they are to make an exhibit of the condition of the science in their respective departments (which latter I presume to be their functions), will be learned, no doubt, at the next meeting, which I shall look forward to with considerable interest.

*Prof. Chapin A. Harris* is gone, and the profession has lost a valuable and a valued member. His reputation as author, teacher and practitioner, is world-wide; and he probably occupied the most exalted position and received more honors than any member of the profession of our day, and deservedly so, for to his labors as an author are to be attributed, more than to any other agency, the rapid advances made by the profession in practice and in the public estimation. Death has removed him from a wide field of usefulness and a host of sincere friends; and those who succeed us in professional life will revere his memory with the same unanimity we now deplore his loss.

*Dr. T. W. Evans* has returned to Paris, after quite a prolonged visit to his American home and friends. While here, he favored a company of professional acquaintance with an exhibition of the various and valuable presents, such as diamond pins, rings, snuff-boxes, insignia of honors, etc., etc., made him by the crowned heads and nobility of Europe, amounting in intrinsic value—so supposed—to the handsome sum of thirty or thirty-five thousand dollars, but upon which he very naturally places a far higher value, as evidences of a splendid success in his profession, and of which he is justly proud.

*Poor Jobson* is in limbo at last. After bullying our mutual friend, Dr. John Allen, and numerous others in New York, and levying black mail wherever there was hope of plunder, he has at last been picked up in London for libeling a distant relative of some distinction, and has been awarded a year's residence at hard labor in one of the English penal establish ments. Yours, O. U. C.

PHILADELPHIA, Oct. 20, 1860.

